# An Essay on the Treatment of Some Affections of the Prostate Gland

**Published:** 1840-10

**Authors:** 


### Art. XIV.

*An Essay on the Treatment of some Affections of the Prostate Gland.* By R. A. Stafford, Surgeon to the St. Marylebone Infirmary.—*London*, 1840. 8vo, pp. 86.

The object of the present brief essay is to direct the attention of the profession to a *new mode* of treating some of the affections of the prostate gland, and to state the experience of the author regarding it. Unimportant as this piece of structure seems to the animal economy in its normal condition, it is nevertheless productive of much misery under the influence of morbid action. Its diseases are generally tedious and difficult of removal; and, even when not immediately threatening the destruction of life, fill the mind of the sufferer with anxiety and apprehension. We are at all times ready to hail the appearance of any work wherein "the old beaten path" is deserted, and something brought forward which at once claims our respectful attention, and promises to open up a field of future utility.

Above all, we are captivated by a strictly practical work, where we find facts of the most interesting kind faithfully narrated, and from which conclusions the most important can be fairly deduced.

Mr. Stafford has long been favorably known to his professional brethren for the skill which he has displayed in overcoming, by artificial contrivances, some of the diseases of the urinary organs; and although we may not be inclined to fall into some of his views, or adopt all his plans of treatment, we cannot refuse our tribute of praise to his zealous exertions. In the short essay before us, he has confined his remarks to chronic enlargement of the prostate gland, and particularly of its third lobe, and we shall best serve our purpose by presenting our readers with an account of the *new* mode of management which the author has adopted, and for the most part in his own words. After a description of the usual symptoms, and observing that it is in the early and middle stages of the disease that most benefit is to be derived, he proceeds:

"The methods I have employed for the treatment of an enlargement of the third lobe of the prostate gland are, the application of certain substances upon it; puncturing the part with a particular instrument I have invented, and perforating it with the urethral perforator, according, in all cases, to the extent of its increase of size. If the third lobe has only partially enlarged, I have then employed remedies, such as iodine, the iodide of potassium, belladonna, &c. locally, always in a diluted form, and combined or uncombined with other substances as the urgency of the case might require. If it be so large that it will not yield to the application of these substances, I have in some instances punctured it with advantage, and when its volume has been so great as to block up the neck of the bladder and cause retention of urine, I have then been under the necessity of perforating it." (p. 16.)

After remarking that it was the effect of iodine over glandular swellings which led him to think of its employment in enlargement of the prostate, he proceeds:

"I felt it easy to make it to be absorbed through the substance of the prostate gland, by using it in suppositories passed up the rectum, but the difficulty was to apply it on the third lobe without touching any other part of the urethra. I made several attempts to accomplish this; first, through a tube, then in a groove at the end of a solid instrument, then by the method by which caustic is applied to a stricture, according to Ducamp's plan, and others; but none of these answered. I at length thought of a very simple mode of applying it, which is by charging a bougie at its point with the *iodine, iodide of potassium,* or any other substance you may wish, and then dipping it into melted tallow so that a coating may be formed upon it. By such method I have been enabled to introduce any application I might desire up to the prostate gland, without touching the surface of any other part of the urethra. The bougie having reached the desired spot, its point is allowed to rest upon the diseased part, when the tallow gradually melts and brings the iodine or iodide of potassium into contact with it, and by drawing the bougie gently backwards and forwards the necessary friction is produced. I have found it advisable to be very cautious as to the strength of the application, for the prostate gland will not bear a strong preparation either of the iodine or iodide of potassium at first. It is usually in an irritable or inflamed state; consequently, even the mechanical pressure of the bougie will give pain. The preparations I have used therefore have been very mild. At first I have found it necessary to employ even anodynes, such as belladonna, opium, hyoscyamus, &c. to quiet irritation and pain. When these have subsided, I have begun carefully by introducing the iodide of potassium in the proportion of *one grain to the drachm* of *unguentum cetacei,* and increasing it as the patient could bear it. I have then gone on with two, three, four, five, and even as far as *ten* grains, or a *scruple* to the drachm, according as the case required it. After this I have added iodine to it; half a grain, one, two, three, four, or even more grains in the same manner. The surgeon who applies it can alone judge of its effects."(p.19.)

The above extract contains the whole of Mr. Stafford's observations respecting the use and application of iodine in the earlier stages of enlarged prostate gland. They are followed by a detail of cases illustrating still farther the efficacy of this plan of treatment. These cases are also valuable in proving, what we have long known to be true, that enlargement of the prostate is not a change incidental merely to old age, but is met with at all periods of life as a consequence of stricture, gonorrhœa, or other affections of the urinary organs. We do not deny, be it understood, that enlargement is not a common sequel of old age; but we mean to affirm that it is, contrary to general supposition, likewise frequent in young subjects. This is a point of very great consequence to be understood, for we have often been able to mitigate symptoms where, from the age of the individual, stricture alone had been suspected, and, from no such impediment having been found or removed, the case had been abandoned as incurable. We would wish it then to be more universally known that disease of the prostate gland prevails at all periods.

In the *second* place, and without any preliminary explanation, the author details two cases wherein he punctured the enlarged lobe. When the gland is "so large that it will not yield to the application of these substances, (iodine, &c.) I have in some instances punctured it with advantage." We have no experience of such a proceeding, but Mr. Stafford has performed it several times. When the third lobe forms an obstinate valvular obstruction about the neck of the bladder, repeatedly puncturing its substance would seem to have the effect of dissipating the swelling. When the gland is thus punctured, a mucous fluid generally makes its escape, but no hemorrhage or other unpleasant symptoms follow the operation. "Experience alone can show its ultimate utility; but from mere reasoning, the puncturing of an enlarged and hardened part, with the view of reducing it, appears rational, and facts even, as far as they go, lead us to such a conclusion." Whether this theory is sound or not we will not stay to determine ; we shall only observe that the prostate gland when not in a highly inflamed or irritable state may be freely dealt with, and that many surgeons err in their treatment of some cases of retention of urine, from not being sufficiently aware of this circumstance.

In the *third* and last place, the author relates three cases where he perforated the middle lobe of the prostate gland. When the swelling is so great that the neck of the bladder is completely blocked up, and no catheter can be passed onwards into the interior, the patient must be relieved at all hazards, else the bladder will slough or urinary coma will succeed. Under these circunstances, the surgeon is reduced to the alternative of either puncturing the distended organ, or carrying an instrument through the seat of obstruction.

We may here remark that, in the whole course of our own experience, we have not met with one single instance where it was necessary to puncture the bladder on account of disease of the prostate; and we do hope, for the credit of British surgery, that operations in such cases are now done away with. We repeat that puncturing the bladder is unnecessary, and therefore unjustifiable, in enlargement merely of this glandular organ. There is not, or cannot be, an instance where the obstruction may not be overcome by milder and far less dangerous measures. When the lateral portions of the prostate are enlarged, the swelling, although great, is never such as to prevent a catheter of sufficient length and flexibility from entering the bladder. It is when along with this the third lobe is so increased in size as to form a direct mechanical obstruction at the commencement of the urethra that the case assumes a very formidable aspect. Even here the want of success is chiefly attributable to the want of tact on the part of the operator, and especially to the use of an inflexible instrument which cannot accommodate itself to the state of the passage, and is with the utmost difficulty guided onwards by the finger in the rectum. By the use of a small elastic catheter without a stilette, we have succeeded in relieving retention of urine under the most urgent circumstances. We should say, as a general rule, that the greater the swelling, the less chance there is of passing a stiff, curved instrument. If, after the trial of all ordinary expedients, the bladder still remains unrelieved, then the best and safest plan is unquestionably to perforate the obstruction. Our readers, we presume, are aware that this plan did not originate with Mr. Stafford, but has been known and acted on for several years back. The perforation is easily accomplished, and is never followed by disagreeable effects.

Of Mr. Stafford's three modes of treatment we have to remark, that of the first two we can at present say nothing from our own experience; but should they be found as useful in the hands of others as they have been in his own, he will be entitled both to the thanks of his medical brethren and of those who are the subjects of this troublesome disorder. It is a rare thing for us to have to complain of brevity, but in Mr. Stafford's pamphlet the explanation is too short compared with the number of pages which the cases are made to occupy. There is an obscurity and a defect too in some parts of the description which we should wish to see remedied. We are at a loss, for example, to know exactly whether he uses the iodine in substance or made into an ointment; and if, as it would seem, the latter, how, we might ask, does he preserve it from melting when the end of the bougie is dipped into hot tallow? Again, we are told that partial enlargement of the third lobe may be discovered "by passing a catheter immediately after the patient has made water; urine will be left in the bladder, amounting to two, three, four, five, or six ounces, and even more, according to the extent of the increase of its volume." The same thing is found where the body of the gland is itself enlarged, and where the portion which forms the third lobe has not participated in the disease. The bladder being unable to completely empty itself is no proof therefore that the third lobe is affected, unless we have first ascertained, by examination through the rectum, that the rest of the prostate is sound.

We have made these remarks more with the view of drawing the author's attention towards them at a future period, than for the purpose of exhibiting our peculiar faculty for finding faults. The essay is decidedly a practical one, and well worthy of perusal; and we sincerely wish that the experience of the author may be confirmed by others who have the best opportunities of testing its merits.

